# Nonabsorbable Antibiotics Reduce Bacterial and Endotoxin Translocation in Hepatectomised Rats

**DOI:** 10.1155/1997/49681

**Published:** 1997

**Authors:** S. K. Kakkos, J. Kirkilesis, C. D. Scopa, A. Arvaniti, T. Alexandrides, C. E. Vagianos

**Affiliations:** 1 Departments of Surgery University of Patras Medical School Patras Greece; 2 Laboratories of Pathology University of Patras Medical School Patras Greece; 3 Laboratories of Microbiology University of Patras Medical School Patras Greece; 4 lnternal Medicine University of Patras Medical School Patras Greece

## Abstract

There is increasing evidence that septic complications, occurring after major hepatectomies, may be caused by gram negative bacteria, translocating from the gut. We investigated in rats, the effect of extended hepatectomy on the structure and morphology of the intestinal mucosa as well as on the translocation of intestinal bacteria and endotoxins. We also examined the effect of nonabsorbable antibiotics on reducing the intestinal flora and consequently the phenomenon of translocation by administering neomycin sulphate and cefazoline. Hepatectomy was found to increase translocation, while administration of nonabsorbable antibiotics decreased it significantly. In addition, hepatectomy increased the aerobic cecal bacterial population, which normalised in the group receiving antibiotics. Among the histological parameters evaluated, villus height demonstrated a significant reduction after hepatectomy, while the number of villi per cm and the number of mitoses per crypt, remained unchanged. Our results indicate that administration of nonabsorbable antibiotics presents a positive effect on bacterial and endotoxin translocation after extended hepatectomy, and this may be related to reduction of colonic bacterial load as an intraluminal effect of antibiotics.


*HPB Surgery*, 1997, Vol. 10, pp. 283–291
Reprints available directly from the publisher
Photocopying permitted by license only

© 1997 OPA (Overseas Publishers Association)
Amsterdam B.V. Published in The Netherlands
by Harwood Academic Publishers
Printed in India

### Nonabsorbable Antibiotics Reduce Bacterial and Endotoxin Translocation in Hepatectomised Rats

S. K. KAKKOS [a], J. KIRKILESIS [a], C. D. SCOPA [b], A. ARVANITI [c], T. ALEXANDRIDES [d] and C. E. VAGIANOS [a]

[a] *Departments of Surgery,* [b] *Laboratories of Pathology and* [c] *Microbiology,* [d] *Internal Medicine, University of Patras Medical School, Patras, Greece*

*(Received 15 May 1996; In final form 10 September 1996)*

**There is increasing evidence that septic complications, occurring after major hepatectomies, may be caused by gram negative bacteria, translocating from the gut. We investigated in rats, the effect of extended hepatectomy on the structure and morphology of the intestinal mucosa as well as on the translocation of intestinal bacteria and endotoxins. We also examined the effect of nonabsorbable antibiotics on reducing the intestinal flora and consequently the phenomenon of translocation by administering neomycin sulphate and cefazoline. Hepatectomy was found to increase translocation, while administration of nonabsorbable antibiotics decreased it significantly. In addition, hepatectomy increased the aerobic cecal bacterial population, which normalised in the group receiving antibiotics. Among the histological parameters evaluated, villus height demonstrated a significant reduction after hepatectomy, while the number of villi per cm and the number of mitoses per crypt, remained unchanged. Our results indicate that administration of nonabsorbable antibiotics presents a positive effect on bacterial and endotoxin translocation after extended hepatectomy, and this may be related to reduction of colonic bacterial load as an intraluminal effect of antibiotics.**

*Keywords:* Hepatectomy, bacteria, endotoxin, bacterial translocation, antibiotics

### INTRODUCTION

The intestinal mucosa not only has metabolic, endocrine and immunological functions but also serves as a barrier to the translocation of intraluminal bacteria and endotoxins to the systemic circulation, distal tissues and organs [1]. This action of intestinal mucosa depends on its anatomical integrity, immunological efficacy and normal intestinal flora [2]. In various diseases, this intestinal barrier is disrupted, leading to the invasion of bacteria, and their products, such as endotoxins, to extraintestinal tissues, a phenomenon known as "Enteric Bacterial Translocation" (EBT) [3, 4]. There is a steadily growing clinical and experimental interest in EBT and the role of the gastrointestinal tract as a source of infectious bacteria, possibly leading to multiple organ failure and death [5].

Liver surgery is associated with significant morbidity and mortality [6], almost 20% of patients undergoing major liver resections, will

Address for correspondence: C. E. Vagianos, M.D., Department of Surgery, Rion University Hospital, 265 00, Patras, Greece, Tel: +3061-999299, Fax:+3061-993984.

suffer from infectious complications. The frequent finding of intestinal bacteria within inflammatory areas after hepatectomy, implies a role for the intestinal tract in the induction of septic complications [7].

In this study, we examined the effect of extended hepatectomy on the structural and functional integrity of the intestinal mucosa and we investigated whether nonabsorbable antibiotics may have a beneficial effect on the phenomenon of bacterial and endotoxin translocation, probably by normalising the intestinal microflora, which was significantly increased after hepatectomy.

### MATERIALS AND METHODS

Male Wistar rats ($n = 90$), weighting 250–320 gr, were used. The animals were housed in stainless-steel cages (three rats per cage), under controlled temperature (23°C) and humidity conditions, and 12-hour dark/light cycles.

The experimental procedure was approved by the Ethics Committee of our University.

The animals were divided, randomly, into four groups according to the treatment they received: Group I ($n = 21$), nonoperated controls, Group II ($n = 17$), sham hepatectomy, Group III ($n = 26$), hepatectomy and Group IV ($n = 26$), hepatectomy plus antibiotics.

The animals belonging to group IV received neomycin sulphate 20 mg per day (Upjohn) and cefazoline 10 mg per day (Fujisawa Pharmaceutical Co., Ltd). The antibiotics, diluted in 2.5 ml normal saline were administered twice daily, via a nasogastric tube, for 10 days, starting eight days prior to, and continuing 48 hours after surgery. Animals from groups II and III were also gavaged with equal volumes of normal saline. They all had free access to standard laboratory chow and tap water throughout the experiment, except for overnight fasting, before surgery.

### Surgical Technique

Operations on group II, III and IV animals were performed on day 8, under light ether anaesthesia, using sterile techniques. Group II animals underwent laparotomy and mobilisation of the liver, while groups III and IV underwent extended, almost 70%, hepatectomy, as described by Higgins and Andersson [8]. All abdominal incisions were closed in two layers (chromic cat gut 5-0 and silk 5-0). On the 10th day the animals of Group I were operated on and those of groups II, III and IV were reoperated, again under sterile conditions. Blood and tissues were obtained according to the experimental protocol and the animals were then sacrificed by exsanguination.

### Endotoxin Measurement in Portal and Aortic Blood

For the determination of endotoxin concentrations, the portal vein and the abdominal aorta were punctured and one and two ml of blood were obtained, respectively. Blood was collected from 10 animals of Group I, six of Group II, 11 of Group III and 14 of Group IV. Endotoxin concentration was determined by using the Limulus Amebocyte Lysate test (LAL), proposed in 1987 by the Food and Drug Administration (FDA), for the determination of endotoxin in biological materials and medical prostheses [9].

### Mesenteric Lymph Nodes(MLN)/Liver Cultures

The small bowel mesentery, including mesenteric lymph nodes, was excised, homogenised, placed into sterile tubes containing thioglycolate broth and incubated at 37°C. Any aerobic growth was Gram stained and cultured on blood and McConcey's agar for 24 hours at 37°C. Different organisms were identified by using standard microbiologic techniques. Aerobic cultures of liver samples were performed by using the same method.

### Cecal Bacterial Population

Collection of cecal contents for the determination of the bacterial concentration, was performed by the following technique: The ascending colon was ligated and a 21 G needle mounted on a 5 ml syringe was introduced in to the cecum, through the ileocecal valve, after puncturing the terminal ileum. Then, 2 ml of sterile saline were infused in the cecum, the needle was withdrawn and the terminal ileum was ligated. The cecal content was manually massaged for two minutes. After a good mixture was achieved, one ml was collected by puncturing the cecal wall. 0.5 ml of the aspirated material was placed in a sterile 20 ml test tube containing Tryptone Soya Broth (dilution 1/40). This was further diluted and 0.0001 ml were recultured in blood agar and McConcey's agar, for the growth and identification of aerobic bacteria. Coliform units per ml of aerobic Gram (+) and Gram (−) bacteria were counted.

### Histological Analysis

One 2 cm long sample was removed from the terminal ileum, for histologic examination. The specimen was fixed in 10% neutral buffered formalin and embedded in paraffin. Tissue sections of 4 µm thick were stained with hematoxylin-eosin. Under a light microscope, the following morphologic mucosal parameters were examined: number of villi per cm (V/cm), villus height (Vh) in mm and number of mitoses per crypt (M/c).

### Measurement of Mucosal DNA and Protein

DNA and protein were determined in the terminal ileal mucosa in six animals from each group. A sample, one cm long, was obtained, it was opened by longitudinal incision and it was washed with cold normal saline. Then, by using a clean glass slide the mucosa was removed and homogenised in one ml NaOH 1 N, by means of a polytron homogeniser. The protein was measured according to Lowry's method [10], by using a commercial kit (Sigma Diagnostics, Germany) and the DNA determined, according to Burton's method, as modified by Giles and Myers [11].

### Statistics

In order to assess the statistical importance of the observed differences of categorical variables among groups, we employed the Chi-Square test or Fishers' exact probability test, when the smallest expected value was less than 5. Analysis of differences of continuous variables, were made by using the Kruskal-Wallis One-Way Analysis by Ranks. $p$ values less than 0.05 were considered as statistically significant.

### RESULTS

One animal in each of groups II, III and IV died during the operation (1) or postoperatively (2). The remaining 87 animals survived the whole experiment without significant problems.

### Endotoxin Concentrations

Table I depicts the endotoxin levels, measured in portal and aortic blood. It is clearly shown that hepatectomy significantly increased endotoxin in both portal and aortic blood. Although a decrease of the respective values in antibiotic treated animals was observed, only the portal endotoxin levels decreased significantly.

### Mesenteric Lymph Nodes/Liver Cultures

The results of the MLN and liver cultures are shown in Table II. Fifty six percent of hepatectomised animals had positive MLN cultures, and this was significantly higher than in controls and sham hepatectomised. In the antibiotic treated group, positive MLN cultures were found in 28%. This was significantly lower compared to

TABLE I Portal and systemic endotoxin levels (values are expressed as mean ± standard deviation)

| Group (n) | I (10) | II (6) | III (11) | IV (14) |
|---|---|---|---|---|
| **Endotoxin (EU/ml)** | | | | |
| Portal blood (PB) | 0.29±0.25 | 0.22±0.15 | 1.35±0.46 | 0.57±0.4 |
| Aortic blood (AB) | 0.15±0.17 | 0.17±0.15 | 0.55±0.3 | 0.4±0.33 |

PB

I vs III: $p$=0.001,

II vs III: $p$=0.0009,

III vs IV: $p$=0.0008,

(Kruskal-Wallis One-Way Analysis by Ranks)

AB

I vs III: $p$=0.005,

I vs IV: $p$=0.035,

II vs III: $p$=0.016,

TABLE II Positive cultures of Mesenteric Lymph Nodes (MLN) and Liver

| Group (n) | I (21) | II (16) | III (25) | IV (25) |
|---|---|---|---|---|
| Positive cultures *n* (%) | | | | |
| MLN | 3 (14) | 1 (6) | 14 (56) | 7 (28) |
| LIVER | 0 (0) | 1 (6) | 9 (36) | 1 (4) |

*Mesenteric Lymph Nodes:* I vs III: $p$=0.004, II vs III: $p$=0.001, III vs IV: $p$=0.045
*Liver:* I vs III: $p$=0.002*, II vs III: $p$=0.032*, III vs IV: $p$=0.005
*(Chi-Square test or Fishers'exact probability test)

the hepatectomy Group, and did not differ from groups I and II. A significant increase in positive liver cultures from hepatectomised animals was also noted, while in group IV the results were similar to those of groups I and II. All the isolated bacteria of aerobic MLN and liver cultures were of intestinal origin. Positive liver cultures were found only when MLNs were also contaminated and in these cases the isolated bacteria were all identical.

### Aerobic Cecal Bacterial Population

There was a statistically significant increase of the aerobic cecal bacterial population after hepatectomy, but this returned to normal when hepatectomy was combined with administration of nonabsorbable antibiotics (Tab. III).

### Histological Analysis

Table IV depicts the histological findings of the morphologic parameters of the intestinal mucosa. The height of villi was significantly shorter after hepatectomy and did not seem to increase significantly by the administration of antibiotics. The other parameters were not found to differ after hepatectomy, in any group.

### Mucosal DNA and Protein

There were no statistically significant differences, among the four groups, regarding the content in DNA and protein of the ileal mucosa.

### DISCUSSION

It is possible that EBT to regional lymph nodes, represents a natural phenomenon, that promotes stimulation of the immune system [12]. However, the massive lymphogenous or hematogenous spread of intestinal bacteria, observed during the course of several pathologic processes [13], may result in hypermetabolism [14], infections [15], sepsis and multiple organ failure syndrome [16].

TABLE III Aerobic Cecal Bacterial Population in groups II, III and IV (values are expressed as mean ± standard deviation)

| Group (n) | II (7) | III (10) | IV (15) |
|---|---|---|---|
| **aerobic bacteria ($10^6$ cfu/ml)** | 15±14 | 37±20 | 7±8 |

II vs III: $p$=0.04, III vs IV: $p$=0.001
(Kruskal-Wallis One-Way Analysis by Ranks)

TABLE IV Structural parameters of terminal ileal mucosa (values are expressed as mean ± standard deviation)

| Group (n) | villi per cm (number) | villi height (mm) | mitoses per crypt (number) |
|---|---|---|---|
| I (10) | 47±15 | 0.41±0.01 | 0.67±0.55 |
| II (10) | 49±13 | 0.42±0.01 | 0.57±0.4 |
| III (10) | 50±6 | 0.27±0.05 | 0.68±0.4 |
| IV (10) | 52±6 | 0.29±0.07 | 0.65±0.53 |
| $p$ | NS | I vs III $p$=0.001<br>I vs IV $p$=0.001<br>II vs III $p$=0.001<br>II vs IV $p$=0.004 | NS |

(Kruskal-Wallis One-Way Analysis by Ranks)
NS=non significant

The decreased secretion of bile to duodenum, after hepatectomy, and therefore the absence of the trophic, bacteriostatic and endotoxin binding action of bile acids, may lead to mucosal atrophy, intestinal flora overgrowth and increase in intraluminal endotoxin concentration, which may be related to disruption of the mucosal barrier and EBT [17–19]. Apart from diminished bile production and, therefore, compromised local trophic and bacteriostatic action of bile acids, significant dysfunction of hepatic reticuloendothelial system [20] and possible immunosuppresive effects of hepatectomy, have been implicated. Furthermore, delayed intestinal transit, leading to enteric bacterial overgrowth [21], and altered permeability of the cell membrane of enterocytes [22] may contribute.

Our results demonstrate a significant increase of endotoxin in the portal and systemic circulation, after hepatectomy, which was decreased in the group also receiving nonabsorbable antibiotics. It has been reported that in both clinical and experimental jaundice, significant endotoxaemia and EBT [23–25] are found in almost 45% of cases. The cause of endotoxaemia is possibly related to increased intraluminal endotoxin, due to the relative lack of intraluminal bile salts and their endotoxin binding capacity [22], while administration of antiendotoxin agents such as lactulose may reduce endotoxinemia [25]. On the other hand, the increased absorption of intestinal endotoxins along with their decreased clearance from the Kupffer cells, may result in pronounced systemic endotoxaemia in hepatectomised animals [26].

We also found increased translocation of intestinal bacteria after hepatectomy, to MLNs and liver. We did not perform anaerobic cultures, since it is well established that translocating bacteria are almost exclusively aerobic [27]. The MLNs are the first location to be reached by the translocated bacteria and therefore are the more frequently colonised sites, while the liver, as the "next target", is colonised to a lesser, but statistically significant, degree. The fact that the bacteria found in MLNs and liver were of intestinal origin and of the same type, suggests a common route and direction of dissemination. It is reported that the administration of absorbable antibiotics promotes bacterial translocation [28], while oral or intravenously administered ethylhydroxyethyl cellulose, a non-ionic, water soluble derivative of cellulose, prevents bacterial translocation in the rat [29]. Experimental data, of early increase of EBT after hepatectomy, are reported in the literature [30, 31], and EBT seems to be related to the extent of the hepatectomy. A 70% hepatectomy in our study, resulted in an increased number of aerobic bacteria in the colon, probably due to the absence of the bacteriostatic action of intraluminal bile acids. Forty eight hours after the operation the intestinal flora of the animals receiving antibiotics was normal, due to prevention of enteric bacterial overgrowth. An increased number of E. coli in distal small bowel and colon, 12–24 hours after a 70% hepatectomy has also been reported by others [8].

Bile and especially bile acids, apart from their bacteriostatic and antitoxin action, also have a trophic effect on the intestinal mucosa [21, 32] and their absence may result in mucosal atrophy. In our experiment, the decrease in intestinal villus height might be an indication of early intestinal atrophy, not effected by changes in the number of intestinal bacteria. The other examined histological parameters did not differ among the four groups, and this may be due to the short time of the study and, possibly, to the only partial absence of the bile acids.

It has been reported that acute liver failure induced by 90% hepatectomy results in reduction of protein content in enterocytes [33]. We did not observe any reduction in intestinal mucosal protein or DNA content after hepatectomy, possibly because of different mechanisms implicated after hepatectomy.

Gut decontamination has several clinical applications, such as the prophylaxis for infection in intensive care unit patients [34]. While the use of polymyxin B, amikacin and amphotericin B has been reported to reduce the incidence of bacterial translocation and early mortality in mice with experimental acute necrotising pancreatitis [35]. On the other hand, overuse of antibiotics may result in opportunistic gut infection, e.g. Clostridium difficile diarrhea [36], which probably represents a contraindication in their routine use.

Our findings suggest that hepatectomy is followed by increased translocation of intestinal bacteria and endotoxin, a phenomenon which may be related to increase in gut bacterial population, due to the acute reduction of bile and bile acid secretion to intestinal lumen. The protective effect of nonabsorbable antibiotics may be attributed to the decrease of aerobic intestinal flora, the main source of translocating bacteria and endotoxins. This may be of some clinical significance, in reducing posthepatectomy septic complications, that remain the main cause of morbidity in major liver surgery.

*Acknowledgements*

The authors acknowledge the skilful technical assistance of the histology technician R. Rassia.

### *References*

[1] Deitch, E. A. (1990). The role of intestinal barrier failure and bacterial translocation in the development of systemic infection and multiple organ failure, *Archives of Surgery*, **125**, 403–404.

[2] Deitch, E. A. (1990). Gut failure: its role in the multiple organ failure syndrome. In: *Multiple organ failure*, edited by Deitch, E. A., New York, NY: Thieme.

[3] Saadia, R., Schein, M., MacFarlane, C. and Boffard, K. D. (1990). Gut barrier function and the surgeon, *British Journal of Surgery*, **77**, 487–92.

[4] Alexander, J. W., Boyce, S. T., Babcock, G. F., Gianotti, L., Peck, M. D., Dunn, D. L., Pyles, T., Childress, C. P. and Ash, S. K. (1990). The process of microbial translocation, *Annals of Surgery*, **212**, 496–512.

[5] Van Leeuwen, P., Boermeester, M. A., Houdijk, A. P., Ferwerda, C. C., Cuesta, M. A., Meyer, S. and Wesdorp, R. I. (1994). Clinical significance of translocation, *Gut*, 35(1), 28–34.

[6] Iwatsuki, S. and Starzl, T. E. (1988). Personal experience with 411 hepatic resections, *Annals of Surgery*, **208**, 421–434.
[7] Wang, X. D. (1993). Bacterial translocation after major liver resection. Doctoral thesis, Bulletin no 89, Lund University, Lund, Sweden.
[8] Higgins, G. M. and Andersson, R. M. (1931). Experimental pathology of the liver. I. Restoration of the liver of the white rat following partial surgical removal, *Archives of Pathology*, **12**, 186–202.
[9] Friberger, P., Knos., M. and Mellstam, L. (1992). *Endotoxins and their detection with the Limulus Amebocyte Test*, pp. 196–206, New York: AR Liss.
[10] Lowry, O. H., Rosenbrough, N. J., Farr, A. L. and Randall, R. L. (1951). Protein measurement with the folin phenol reagent, *Journal of Biological Chemistry*, **193**, 265–275.
[11] Giles, K. W. and Myers, M. (1965). Improved diphenylamine method for estimation of DNA, *Nature*, **206**, 93–95.
[12] Gianotti, L., Alexander, J. W., Fukushima, R. and Pyles, T. (1993). Reduction of bacterial translocation with oral fibroblast growth factor and sucralphate, *American Journal of Surgery*, **165**, 195–201.
[13] Mainous, M. R., Tso, P., Berg, R. D. and Deitch, E. A. (1991). Studies of the route, magnitude and time course of bacterial translocation in a model of systemic inflammation, *Archives of Surgery*, **126**, 33–37.
[14] Mochizuki, H., Trocki, O., Dominioni, L., Brackett, K. A., Joffe, S. N. and Alexander, J. W. (1984). Mechanism of prevention of postburn hypermetabolism and catabolism by early enteral feeding, *Annals of Surgery*, **200**, 297–310.
[15] Rush, B. F., Jr., Sori, A. J., Murphy, T. F., Smith, S., Flanagan, J. J., Jr. and Machiedo, G. W. (1988). Endotoxinemia and bacteremia during hemorrhagic shock. The link between trauma and sepsis? *Annals of Surgery*, **207**, 549–554.
[16] Carrico, C. J., Meakins, J. L., Marshal, J. C., Fry, D. and Maier, R. V. (1986). Multiple organ failure syndrome, *Archives of Surgery*, **121**, 196–208.
[17] Bertok, L. (1977). Physio-chemical defence of vertebrate organisms: The role of bile acids in defence bacterial endotoxins, *Perspectives in Biological Medicine*, **21**, 70–76.
[18] Levi, A. C., Borghi, F., Petrino, R., Bargoni, A., Fronticelli, C. M. and Gentilli, S. (1991). Modifications of the trophism of intestinal mucosa after intestinal and biliopancreatic diversion in the rat, *Italian Journal of Gastroenterology*, **23**, 202–207.
[19] Cahill, C. J., Pain, J. A. and Bailey, M. E. (1987). Bile salts, endotoxin and renal function in obstructive jaundice, *Surgery, Gynecology and Obstetrics*, **165**, 519–522.
[20] Andersson, R. (1990). Infection in hepato-pancreaticobiliary surgery. In *Surgery of the liver and biliary tract*, edited by Blumgart, L. H., pp. 147–160. Churchil Livingstone.
[21] Wang, X. D., Guo, W. D., Wang, Q., Andersson, R., Ekblad, E., Soltesz, V. and Bengmark, S. (1994). The association between enteric bacterial overgrowth and gastrointestinal motility after subtotal liver resection or portal vein obstruction in rats, *European Journal of Surgery*, **160**, 153–160.
[22] Wang, X. D., Parsson, H., Andersson, R., Soltesz, V., Johansson, K. and Bengmark, S. (1994). Bacterial translocation, intestinal ultrastucture and cell membrane permeability early after major liver resection in the rat, *British Journal of Surgery*, **81**, 579–584.
[23] Deitch, E. A., Sittig, K., Li, M., Berg, R. and Specian, R. D. (1990). Obstructive jaundice promotes bacterial translocation from the gut, *American Journal of Surgery*, **159**, 79–84.
[24] Pain, J. A. and Bailey, M. E. (1987). Measurement of operative plasma endotoxin levels in jaundiced and non-jaundiced patients, *European Surgical Research*, **19**, 207–216.
[25] Vagianos, C., Koureleas, S., Arvaniti, A., Kirkilesis, J., Scopa, C. D., Dervenis, C. and Stavropoulos, M. (1995). Bacterial translocation and endotoxinemia in obstructive jaundice: the protective effect of lactulose. *Digestive Surgery*, **12**, 321–326.
[26] Pain, J. A. (1987). Reticulo-endothelial function in obstructive jaundice, *British Journal of Surgery*, **74**, 1091–1094.
[27] Steffen, E. K., Berg, R. D. and Deitch, E. A. (1988). Comparison of translocation rates of various indigenous bacteria from the gastrointestinal tract, *Journal of Infectious Diseases*, **157**, 1032–1038.
[28] Deitch, E. A., Maejima, K. and Berg, R. (1985). Effect of oral antibiotics and bacterial overgrowth on the translocation of the GI tract microflora in burned rates, *Journal of Trauma*, **25**, 385–392.
[29] Wang, X., Andersson, R., Soltesz, V., Guo, W. and Bengmark, S. (1993). Water-solube ethylhydroxyethyl cellulose prevents bacterial translocation induced by major liver resection in the rat, *Annals of Surgery*, **217**, 155–167.
[30] Wang, X., Andersson, R., Soltesz, V. and Bengmark, S. (1992). Bacterial translocation after major hepatectomy in patients and rats, *Archives of Surgery*, **127**, 1101–1106.
[31] Wang, X. D., Soltesz, V., Andersson, R. and Bengmark, S. (1993). Bacterial translocation in acute liver failure induced by 90 per cent hepatectomy in the rat, *British Journal of Surgery*, **80**, 66–71.
[32] Granger, D. N., Richardson, P. D., Kvietys, P. S. and Mortillaro, N. A. (1980). Intestinal blood flow. *Gastroenterology*, **78**, 837–863.
[33] Wang, X. D., Ar' Rajab, A., Andersson, R., Soltesz, V., Wang, W., Svensson, M. and Bengmark, S. (1993). The influence of surgically induced acute liver failure on the intestine in the rat, *Scandinavian Journal of Gastroenterology*, **28**, 31–40.
[34] Reidy, J. J. and Ramsay, G. (1990). Clinical trials of selective decontamination of the digestive tract: review, *Critical Care Medicine*, **18**, 1449–1456.
[35] Gianotti, L., Munda, R., Gennari, R., Pyles, T. and Alexander, J. W. (1995). Effect of different regiments of gut decontamination on bacterial translocation and mortality in experimental acute pancreatitis, *European Journal of Surgery*, **161**, 85–92.
[36] McFarland, L. V. (1995). Epidemiology of infectious and iatrogenic nosocomial diarrhea in a cohort of general medicine patients, *American Journal of Infectious Control*, **23**, 295–305.

### INVITED COMMENTARY

**Roland Andersson**

"Non-absorbable antibiotics reduce bacterial endotoxin translocation in hepatectomised rats"

by Kakkos SK, Kirkilesis J, Scopa CD, Arvaniti A, Alexandrides T, Vagianos CE. HPB Surgery 1996 (manuscript no 310).

Septic complications, frequently caused by bacteria of enteric origin, still represent a major part of morbidity following for example extensive liver resections. This is still true despite improvements in surgical technique, antibiotic therapy and intensive care [1]. The gut has been recognised as a major source of these complications and potentially also the development of multiple organ dysfunction [2]. This has emphasized the importance of the intestinal mucosal barrier as a first line of defense against invasion of for example luminal bacteria and toxins [3]. Experimental liver resection is a good and standardized model for studying the complexity of underlying pathophysiological mechanisms in the failure of the intestinal barrier. The present study gives further evidence to the existence of failure of the intestinal barrier with concomitant bacterial translocation and leakage of endotoxin following a major abdominal challenge, in this instance experimental 70% hepatectomy in the rat. Furthermore, the authors also demonstrate the efficacy of non-absorbable antibiotics which reduce the translocation of both bacteria and endotoxin in animals subjected to 70% hepatectomy. This illustrates the potential use of antibiotics in preventing bacterial translocation, but also gives fuel to the more selective use of antibiotics, like selective gut decontamination. It is thus to hope that this elegant study is to be followed up by additional experiments from the group in Patras.

### References

[1] Andersson, R., Saarela, A., Tranberg, K. G. and Bengmark, S. (1990). Intraabdominal abscess formation after major liver resection, *Acta. Chir. Scand.*, **156**, 707–710.

[2] Deitch, E. A. (1992). Multiple organ failure – pathophysiology and potential future therapy, *Ann. Surg.*, **216**, 117–134.

[3] Wang, X. D. and Andersson, R. (1994). Intestinal brushborder membrane function (review), *Scand. J. Gastroenterol.*, **29**, 289–299.

Dr. R. Anderson
Lund University
Lund
S-221 85
SWEDEN

### COMMENTARY ON MANUSCRIPT – NON ABSORBABLE ANTIBIOTICS REDUCE BACTERIAL AND ENDOTOXIN TRANSLOCATION IN HEPATECTAMISED RATS

This paper investigates the concept of bacterial translocation following hepatectomy. Bacterial translocation is not a new concept and has been very extensively investigated. It does seem to be of some clinical significance in patients with sepsis, those with major trauma, those having undergone major surgery or those with obstructive jaundice. This study involves a relatively simple model of extended hepatectomy in the rat and the parameters measured included villous height and number in the jejunum in addition to measurement of portal and systemic endotoxine concentration, estimation of caecal bacterial population and assessment of translocation to the mesenteric lymph nodes. The study demonstrated a reduction in villous height in addition to increased anerobic caecal bacterial counts, increased bacterial translocation and increased portal and systemic endotoxaemia, following hepatectomy. The increased caecal bacterial counts, the increased bacterial translocation and increased portal endotoxaemia were all reversed by enteral administration of non-absorbable antibiotics.

These results are relatively similar to results for increased enteral growth of bacteria and increased translocation and endotoxaemia after other major insults such as trauma, burns or sepsis. The authors quite rightly mention the significant incidence of septic complications after major surgery, such as hepatectomy, and suggest that this may be reduced in the clinical setting by the use of nonabsorbable antibiotics. However, there is a major difference between the laboratory research situation and the actual clinical setting and potential advantages of treatment such as use of nonabsorbable antibiotics, are often not observed when investigated in the clinical setting. There is always a risk when extrapolating the results of laboratory experiments to the clinical setting but the authors quite rightly point out that this is only a theoretical advantage which perhaps should be investigated in the clinical situation.

Before therapy such as this could be adopted in routine hepato-biliary practice, particularly liver resection and surgery for obstructive jaundice, much more work has to be done in the clinical area and some major concerns about the widespread use of antibiotics, such as the development of resistant strains, need to be further investigated and addressed.

Mr. T Diamond
Consultant Surgeon
Mater Hospital
Crumlin Road
BELFAST BT14 6AB
Northern Ireland

